# Cases Illustrative of the Protective Influence of Vaccination against the Contagion of Small-Pox

**Published:** 1855-01

**Authors:** James M. Newman


### ART. III.—*Cases Illustrative of the Protective Influence of Vaccination against the contagion of Small-Pox.* By JAMES M. NEWMAN, M. D.

The following cases are given, not with an idea that they contain within themselves any new facts, or differ in their results from what has probably fallen under the observation of every physician who has come much in contact with variola; but as affording corroborative testimony of the power of vaccination to protect the individual when performed after an exposure to the contagion, a question not unfrequently mooted; and to supply additional evidence of the incalculable value of the discovery and labors of Jenner.

Although there seems to be a pretty general acquiescence in the value of the immunity afforded by this means against a most loathsome and fatal disease, there is, notwithstanding, either much scepticism upon the subject, or positive carelessness upon the part of the public in availing themselves of its benefits; or else, it is fair to presume, the disease would ere this have disappeared from off the face of the earth, or at least disappeared from among civilized men, with their sanitary laws and police.

I have sometimes thought that I have perceived a tendency in physicians, unintentional, perhaps, but none the less pernicious, to depreciate the value of this protection, basing their conclusions upon the occasional and exceptional failure of some case under their observation, and which they are at the pains to give the widest publicity through the medical press.

Exceptions, I believe, occur to all general rules, and probably in these cases, are the results in the great majority of cases of the imperfection of the operation, and the depreciation of the virus by age, and of the substitution of a spurious for a genuine vesicle for the evidence of its success, a result, probably, most frequently the consequence of unprofessional parties deciding upon its character, and being too often satisfied with any species of *sore* where the virus was inserted, without further reference to the physician in the matter.

While there seems abundant reason to believe that either the intensity of the protection is weakened by the lapse of years, and the increasing age of the subject, or that a single vaccination performed in infancy is not in all cases capable of furnishing entire immunity throughout a long life, the public should be taught that as a matter of *prudence*, at least, an occasional re-vaccination should be resorted to, and it should be impressed upon every one as a duty, and as the only means of obtaining entire exemption from all risks of exposure.

The duty of the authorities, state and municipal, to enforce the vaccination of our population by statuary enactments and penalties, should be more prominently and constantly urged upon the attention of the public than is now the custom. No child should be permitted to enter any of our public schools without having submitted to this operation; nor should any person be admitted into any institution, educational, charitable or correctional, without being insured as far as it is possible from all risks of the disease. A more perfect supervision should be exercised over the emigrant ships landing at our seaports. The seeds of the disease are daily being scattered over the country by this class of persons, and perhaps furnish the principal source from which our population at this day suffer. In this city more than three-quarters of all the cases are furnished from, or through the influence of this class. Doubtless other cities to the west suffer in the same manner, as it seems to be the plan of parties interested, to hurry the newly arrived emigrants, with all their filth and disease, from beneath their eyes upon the sea-board, to the distant west, regardless of human suffering, so long as their pecuniary interest is advanced thereby.

The following cases are given as illustrative of the protection afforded by vaccination when performed for the first time after an exposure to small-pox, and the consequent risk of infection incurred.

In February last I was called to visit a German emigrant family, who had been but a short time in the city, and were believed to have contracted the disease on ship-board. I found in a small room, and in very destitute circumstances, a family consisting of the father, mother, and four children, the youngest yet nursing. The mother was covered with a copious crop of variolous vesicles, their number constituting a case of more than usual severity. She had been some eight days sick, and this was about the fourth day of the eruption.

The father and the children, as they told me, had never been vaccinated.

I accordingly vaccinated them, together with another man and his wife who occupied the room with them.

The first family all had, in due course of time, genuine vaccine vesicles, with the exception of one boy about six years old. An examination of the boy revealed the marks of a previous vaccination upon the other arm than the one I vaccinated.

The protection afforded to the inmates of this room, at this lapse of time after their continuous exposure, is as follows:

On the eighth day after the vaccination, the father had two well-developed vesicles upon the arm. On the second day succeeding, or ten days after the vaccination, I found him, on a visit to the house, walking about the room, with a variolous eruption scattered over the face. The vesicles were very few and sparse indeed. I removed him, with his children, to the hospital, more as a measure of sanitary precaution, and to relieve their destitute contion than to furnish medical aid; for he suffered so little inconvenience from the varioloid, that had he not had some inflammation of one eye, he would have required no medication whatever.

The children all escaped the disease entirely except the babe, who must be supposed to have imbibed the disease in its most concentrated form from its mother's breast, and who had about half a dozen moderate sized vesicles upon the face, and a few in its mouth and throat. None could be found upon its body. The vaccine vesicle had not yet completely dried upon the arm at the time.

The two other occupants of their room mentioned above, escaped entirely.

Upon the opposite side of the street from which they were removed, in a small house and among the above party's ship companions, occurred about the same time a case of varioloid. When I first saw the man, the case though not severe was well developed. I removed him to the hospital. The room was occupied by some seven or eight other persons, men, women, and children. I vaccinated them all, some being primary and others secondary vaccinations. Upon visiting the house four or five days afterward, found one of the women had been confined in the interval, and if she did not occupy the same bed from which the case of varioloid had been taken, she at least was upon the same bedstead, and in the same corner of the room. I immediately vaccinated the babe. All the inmates of this room experienced entire immunity from the disease.

A stout healthy German woman, aged twenty-two years, entered the small-pox hospital in June last, as a servant. She had no fears of the disease, but I deemed it prudent to revaccinate her, which I did on the day after her entry, inserting the virus in two places in the left arm. She had upon this arm two or three very distinct and large vaccine scars, the results of a vaccination when a child. These were very distinct, each as large as a sixpence, and presenting that striated appearance which I think every physician must have noticed as being peculiar to the Germans, apparently depending upon the manner of introducing the virus. These marks were so distinct that I might have been justified in relying upon them for protection, and in not performing the operation again. On examining the arm on the ninth day thereafter, I was surprised to find two well-developed vaccine vesicles, with their areola. One of the vesicles was larger than the other. She has remained in the hospital, in perfect health, up to this time.

I think this case is illustrative of the liability of the protective power of the vaccination to be exhausted by age, and of the sufficiency of a revaccination to restore it. Had dependence been placed upon the distinctness of the vaccine scars, as is too often done, and the operation had not been successfully repeated, and the woman suffered from her exposure to the disease, the case would undoubtedly have gone to swell the number of parallel instances written down against the infallibility of this invaluable public boon.

Upon assuming charge in January last of the small-pox hospital, I found the hospital in the keeping of Mr. S. and wife, who had then discharged that duty for the previous fourteen months. Mrs. S. was now in an advanced period of gestation.

For three or four months of the winter no patients had been in the hospital. On the third of February, the first patient of my year of service, was sent there with the small-pox, and a few days more served to fill the wards, the German emigrants furnishing a supply of patients. I felt no little anxiety as to the effect this exposure might have upon Mrs. S., or rather upon the fœtus in utero, remembering the cases on record, where the child has been born covered with variola. These fears I, however, did not communicate to Mrs. S. She had never had the small-pox, and was only protected by vaccination.

On the 26th of February, at a time when the hospital was well filled with patients, labor supervened, and a fine healthy male infant was born. The birth took place about seven in the morning, and at three o'clock the same afternoon I vaccinated him, inserting the virus in the arm in two places. Any unnecessary exposure was guarded against, but complete isolation in such a place was of course impracticable. The virus at one of the points of insertion did not work, but the other produced a well-developed vaccine vesicle, affording complete protection to the child. After the maturing of the pustule and the convalescence of the mother, its exposure was as complete as the rest of the inmates, being carried without reserve in and out of the wards. It has enjoyed universal good health up to this time.

It may not be uninteresting to note here, that Mrs. S. had had, previous to assuming the charge of the hospital, two premature births in succession, occurring at about the seventh month; so that there would seem not to have been wanting any susceptibility to disturbing agencies, nervous or otherwise.

Five years ago a man walked to my office, from a considerable distance, to inquire of me the character of an eruption which had made its appearance two or three days previous, with so little constitutional disturbance as to have almost escaped his notice. The disease was distinctly varioloid. The next morning his wife, the mother of several children, brought me her babe, about six months old, to vaccinate, but declined submitting to the operation herself, assigning as a reason, that she had, when a girl, contracted the vaccine disease directly from the cow, and that her physician at the time told her she need have no fears of the disease, as she was effectually guarded against it. Her other children had all been quite recently vaccinated, and the operation was not repeated. In about a week she was taken down with a smart attack of varioloid, but none of the children suffered from their exposure, though they were not separated an hour from the mother, their recent vaccinations furnishing complete protection.

In the boarding house from which case No. 14 of the following table was removed, every inmate of the house was exposed to the disease, as the case was well developed before his removal to the hospital, and the patient had been permitted to occupy the common sitting-room of the house. All exposed were revaccinated, and none suffered any in consequence of their risk of infection.

The following cases are given as illustrative of the length of time during which the modifying influence of the vaccination extended, but was not sufficient to protect entirely, or had so far become weakened by the lapse of years as not to secure entire immunity from the effects of exposure to the disease. They may also serve to aid in solving the question so often propounded to the physician, "How many years does it take for the vaccination to run out?"

They are extracted from my hospital records, those cases only being given where there seemed undoubted evidence of vaccination having been performed. Some of my records are without any reference to this fact, the inquiry having occasionally been omitted, and in other instances the answers were of so uncertain a character as not to merit preservation. Among the Germans, who have furnished a large portion of the cases under my observation, revaccination seems seldom to be performed. In the land of their nativity vaccination is enforced by law, and is performed in infancy, and upon the security thus afforded them they remain content during life.

| | Name. | Aged. | Length of time since vaccination. | REMARKS. |
|---|---|---|---|---|
| 1 | Mr. L. B., | 35 | 20 years. | Moderate. Discharged on the 21st day from hospital. |
| 2 | " J.G.S., | 29 | 25 " | Very mild. Discharged on the 17th day from hospital. |
| 3 | " C. H., | 34 | childhood. | Very mild. |
| 4 | " J. L., | 34 | " | Moderate. |
| 5 | " C. S., | 33 | " | Very mild. |
| 6 | T. D., | 10 | | Very mild. Had not over 12 vesicles on face. |
| 7 | F. R., | 33 | childhood. | Very mild. Had previously been sick six weeks with typhoid fever. |
| 8 | C. S., | 26 | " | Light. |
| 9 | Mr. H. R., | 26 | " | Severe. Had slight œdema of face, with some vesicles confluent. Body covered with vesicles. |
| 10 | " H. B., | 28 | " | Mild. |
| 11 | J. E. D., | 20 | 15 years. | Vesicles did not run the usual course of suppuration, but seemed to dry up and scale off. |
| 12 | Miss E. B., | 11 | 9 years. | Very mild. Was not confined to the bed. |
| 13 | L. S., | 16 | 10 " | Very mild. Was not confined to the bed. |
| 14 | M. B., | 25 | childhood. | Moderately severe. Had slept three nights in the same room, and in the same bed, which had been occupied by a small-pox patient, but not until a week after the removal of the patient. Was revaccinated twice after exposure, but the virus produced but little influence on his arm. |
| 15 | P. E. M., | 17 | childhood. | Moderately severe. |
| 16 | Miss E. H., | 24 | " | Had varioloid lightly, but had also with it a copious eruption of pemphigus. |
| 17 | " B. S., | 28 | " | Had a copious crop of pustules over the face and body. |
| 18 | G. S., | 22 | " | Very light. |
| 19 | C. D., | 26 | " | Very light. |
| 20 | Miss M. K., | 21 | " | Very light. |
| 21 | J. S., | 36 | " | Had a copious crop of pustules. |
| 22 | Miss B. E. | 24 | | Said she was vaccinated when a child, and again at the age of 15 years, but I could find no marks on the arm. The eruption was copious; considerable œdema of face; much sore throat, and great irritability of stomach. Was discharged in four weeks from date of attack. |
| 23 | Jno. B.W., | 22 | | Moderate. Says he has been vaccinated two or three times on each arm: once when a babe, and the last time eight years since. Thinks they never produced any effect. Can find no marks on his arm, thinks they were there until within a year or two. |
| 24 | H. B., | 24 | childhood. | Had the disease lightly. |

It will be observed, upon examining the above cases, that a number of years have elapsed since the vaccination to the contraction of the disease, but that the periods are too irregular to admit of deducing therefrom any standard in years, at which the protective influence takes its departure, unless it be the general fact of the greater portion of the patients having arrived at adult age. This excess occurs in practice, and is indicative of the general deterioration by age, without our power, perhaps, of ever being able to define definitely the period up to which it is safe to rely upon a single vaccination. This immunity can undoubtedly be purchased by a revaccination, and that too, probably, without the virus producing a genuine vaccine vesicle in return. I have seen several instances of genuine secondary vesicles being obtained where intervals of from twenty-five to thirty years have elapsed between the vaccinations.

There is much difficulty, as any one will find who undertakes the task, in obtaining reliable facts as to the effects, and the character of the vesicles following revaccinations in adults. If the parties deem the matter one of sufficient importance to submit their arm to your examination, it most frequently happens that they have carelessly so broken the vesicles, and marred their appearance that their definite character cannot be made out.

